# Survivin Is Required for Beta-Cell Mass Expansion in the Pancreatic Duct-Ligated Mouse Model

**DOI:** 10.1371/journal.pone.0041976

**Published:** 2012-08-01

**Authors:** Xiaohong Wu, Qinfeng Zhang, Xiaojing Wang, Jiayu Zhu, Kuangfeng Xu, Hitoshi Okada, Rennian Wang, Minna Woo

**Affiliations:** 1 Department of Endocrinology, The First Affiliated Hospital with Nanjing Medical University, Nanjing, China; 2 Division of Signaling Biology, Ontario Cancer Institute, Toronto, Ontario, Canada; 3 Department of Physiology and Pharmacology, Children’s Health Research Institute, University of Western Ontario, London, Canada; 4 Toronto General Research Institute, Toronto, Ontario, Canada; 5 Department of Medicine and Medical Biophysics, University of Toronto, Toronto, Ontario, Canada; 6 Division of Endocrinology and Keenan Research Centre in the Li KaShing Knowledge Institute, St. Michael’s Hospital, Toronto, Ontario, Canada; University of British Columbia, Canada

## Abstract

**Aims/Hypothesis:**

Pancreatic beta-cell mass expands through adulthood under certain conditions. The related molecular mechanisms are elusive. This study was designed to determine whether surviving (also known as Birc5), which is transiently expressed perinatally in islets, was required for beta-cell mass expansion in the pancreatic duct-ligated mouse model.

**Methods:**

Mice with beta cell–specific deletion of *survivin* (RIPCre^+^survivin^fl/fl^) and their control littermates (RIPCre^+^survivin^+/+^) were examined to determine the essential role of survivin in partial pancreatic duct ligation (PDL)-induced beta-cell proliferation, function and survival.

**Results:**

Resurgence of survivin expression occurred as early as day 3 post-PDL. By day 7 post-PDL, control mice showed significant expansion of beta-cell mass and increase in beta-cell proliferation and islet number in the ligated tail of the pancreas. However, mice deficient in beta-cell survivin showed a defect in beta-cell mass expansion and proliferation with a marked attenuation in the increase of total islet number, largely due to an impairment in the increase in number of larger islets while sparing the increase in number of small islets in the ligated tail of pancreas, resulting in insufficient insulin secretion and glucose intolerance. Importantly however, beta cell neogenesis and apoptosis were not affected by the absence of survivin in beta cells after PDL.

**Conclusions/Interpretation:**

Our results indicate that survivin is essential for beta-cell mass expansion after PDL. Survivin appears to exhibit a preferential requirement for proliferation of preexisting beta cells.

## Introduction

As type 1 and type 2 diabetes result from absolute or relative deficiencies in beta-cell mass, respectively, understanding how beta-cell mass is regulated can lead to new therapeutic options. Beta cells replicate slowly after birth [1], but proliferation can be stimulated under various conditions, including obesity, pregnancy and in response to growth factors [2]. However, the mechanism by which new beta cells are formed in adults is still controversial. Genetic lineage tracing studies provide evidence that pre-existing beta cells, rather than stem/progenitor cells, are the major source of new beta cells in adult mice both under normal physiological conditions and after 50% to 70% pancreatectomy [3], [4]. However, a recent study [5] in mice has shown that new beta cells are generated from facultative adult progenitor cells (neogenesis) in response to pancreatic injury, in a manner reminiscent of embryonic endocrine cell differentiation.

Survivin (also known as Birc5) is the smallest member of the inhibitor of apoptosis protein (IAP) gene family [6]. Initially, survivin was described as an anti-apoptosis gene [7]. However, further studies have revealed that survivin is a multifunctional protein that intersects fundamental networks of cellular processes, including cell death, cell division and cellular adaptation [8]–[10]. Germline deletion of survivin leads to early embryonic lethality at E3.5 [11]. Tissue-specific knockout of survivin in thymocytes, neuronal precursors, endothelial cells or haematopoietic progenitors resulted in impaired cell proliferation, cell cycle arrest, apoptosis, or mitotic spindle formation, illustrating a complex physiological role for survivin in normal cell development [12]–[15].

Previous studies have shown that survivin is expressed in beta cells of fetal human pancreas,but not in adult islets [16], [17]. However, survivin is also found in the beta cells in areas of chronic pancreatitis and within lobular areas of surviving islets of pancreata of patients with presumed type 1A (immune-mediated) childhood-onset diabetes [18], [19]. We previously showed that transient perinatal expression of survivin is essential for pancreatic beta-cell mass establishment by regulation of cell cycle progression [20]. Specific deletion of survivin in beta cells does not affect embryonic pancreas or beta-cell development but does impair beta-cell mass expansion, resulting in progressive diabetes in adult male mice [20], [21]. However, female mice remain glucose tolerant despite relatively reduced beta-cell mass and thus are the focus of this study, allowing for analysis of adult beta-cell expansion without the confounding effects of pre-existing hyperglycemia.

To determine whether survivin is required for beta-cell expansion in adults, we performed partial pancreatic duct ligation (PDL) on adult female RIPCre^+^survivin^fl/fl^ mice and control (RIPCre^+^survivin^+/+^) littermates. Here, we show that survivin, which was absent in adult pancreas was induced within beta cells in the ligated tail of pancreas following PDL during the period of peak regeneration, and that expansion of beta-cell mass following PDL was impaired in RIP-Cre^+^survivin^fl/fl^ mice due to reduced beta-cell proliferation.

## Methods

### Mice

Survivin^fl/fl^ mice [12], were mated with mice carrying the Cre transgene under the control of the rat insulin 2 promoter [TgN(ins2-cre)25 Mgn, hereafter referred to as RIPcre; Jackson Laboratories]. RIPcre^+^survivin^+/fl^ mice were intercrossed to generate RIPcre^+^survivin^+/+^, RIPcre^+^survivin^+/fl^ and RIPcre^+^survivin^fl/fl^ mice. Only wild-type (RIPcre^+^survivin^+/+^) littermates were used as controls. Genotypes for the *cre* and *survivin* genes were determined by PCR using mouse tail DNA. PCR primers for survivin (GeneID:11799) were as follows: forward 5′-TGAGTCGTCTTGGCGGAGGTTGT-3′, reverse 5′-GCTCGTTCTCGGTAGGGCAGTGG-3′. All mice were maintained on C57BL/6 background, housed in a pathogen-free facility on a 12-hr light-dark cycle, and fed ad libitum with standard irradiated rodent chow in accordance with Ontario Cancer Institute Animal Care Facility Protocol, without restriction on animal activity. Animal care and experimental protocols were approved by the Nanjing Medical University Animal Care Committee.

### Surgical Procedure and Tissue Preparation

Two-month-old female mice were anesthetized using isoflurane, and laparotomy was performed through a midline abdominal incision. Pancreatic ducts of the gastric and splenic segments were ligated as described [22]. Partial PDL was carefully made from the point where the pancreas is attached to the first portion of the duodenum to the point just before its attachment to the transverse colon. The ligated tail portion accounts for 50–60% of the entire pancreas. For sham operations, the pancreas was gently touched with a moistened cotton-tipped swab for 1 min. Pancreatic head and tail portions were harvested at 3, 7 and 14 days of post-surgery. These specimens were processed separately for weight determination, prior to fixation and embedding in paraffin.

### Metabolic Studies and Hormone Measurements

All overnight-fasts were carried out between 6 pm to 8 am. Blood glucose levels were determined with mouse tail venous blood using an automated glucometer (Roche, Germany). Intraperitoneal glucose tolerance tests (IPGTT) and glucose-stimulated insulin secretion (GSIS) measurements were performed at day 7 post-surgery as previously described [23]. Serum insulin levels were measured by ELISA with rat/mouse insulin standards (Linco, USA).

### Islet Isolation

Pancreatic islets were isolated from 2-week-old mice and ligated tail portion of the mice at day 7 post-surgeryby injecting collagenase V (2 mg/ml; Sigma-Aldrich, USA) into the pancreas. Pancreata was then allowed to digest in the collagenase solution at 37°C with shaking for 15–20 min. Digestion was stopped by ice-cold Hanks’ balanced salt solution (HBSS) containing 10% (vol./vol.) FCS. Islets were then hand-picked under a dissecting microscope.

### Western Blotting

Islet-, liver- and kidney-protein lysates were obtained as previously described [24]. Lysates were separated by SDS-PAGE and immunoblotted with antibodies against survivin (Novus Biologicals, USA), p21 and caspase-3 (BD Pharmingen, USA), Aurora Bkinase (Abcam, USA), v-akt murine thymoma viral oncogenes (AKT), phosphorylatedAKT (Ser473), cleaved caspase-3 (Asp175) and glyceraldehyde phosphate dehydrogenase (GAPDH) (Cell Signaling Technology, USA). Densitometric quantification of protein bands was analyzed using Image J software version 1.37. Samples were normalized to GADPH.

### Immunohistochemistry, Immunofluorescence Staining, and Islet Morphometry

Pancreata were fixed in 4% paraformaldehyde. Samples were dehydrated and prepared as paraffin blocks. Serial seven µm-thick pancreatic sections were obtained with at least 3 levels per slide separated by 100–150 µm intervals and stained with hematoxylin and eosin, insulin (DAKO) and survivin. Beta-cell proliferation was assessed by Ki67 (Novus Biologicals, USA) and apoptosis was detected using In Situ Cell Death Detection Kit (Roche, USA). Double immunofluorescence staining was performed for insulin with survivin or glucagon (NovoCastra Laboratories) and slides were examined using a Zeiss inverted fluorescent microscope. Total beta-cell mass at two month of age and day 7 after PDL were determined on insulin-immunostained sections and calculated by multiplying the pancreatic weight by the ratio of beta-cell area to total pancreatic area. Islet number and beta-cell number and size were calculated on insulin-immunostained sections. According to the nucleus numbers, islets were divided into small islets (1–10 nuclei) and larger islets (>10 nuclei).

### Quantitative Real-time PCR

Total RNA was isolated from ligated pancreas tail at day 7 post-surgery with TRIzol reagent (Invitrogen Life Technologies Inc.,Carlsbad, CA, USA). The cDNA was synthesized from 1 µg RNA using M-MLV reverse transcription system (Promega, Madison, WI, USA). Primers used are listed in Table S1. Real-time RT-PCR analyses were performed using SYBR Green Supermix kit in ABI Prism 7900 HT real-time PCR system (Applied Biosystems, Foster City, CA, USA). The relative levels of gene expression were normalized by the internal control GAPDH. Each sample was run in triplicate, and each experiment included three nontemplate control wells.

### Statistical Analysis

Data are presented as means ± SE and were analyzed by unpaired student’s *t*-test and one-way analysis of variance with the post-hoc Tukey test. Differences were considered to be statistically significant when P<0.05.

## Results

### PDL Induces Survivin Expression and Promotes Beta-cell Mass Expansion

**Figure 1:**
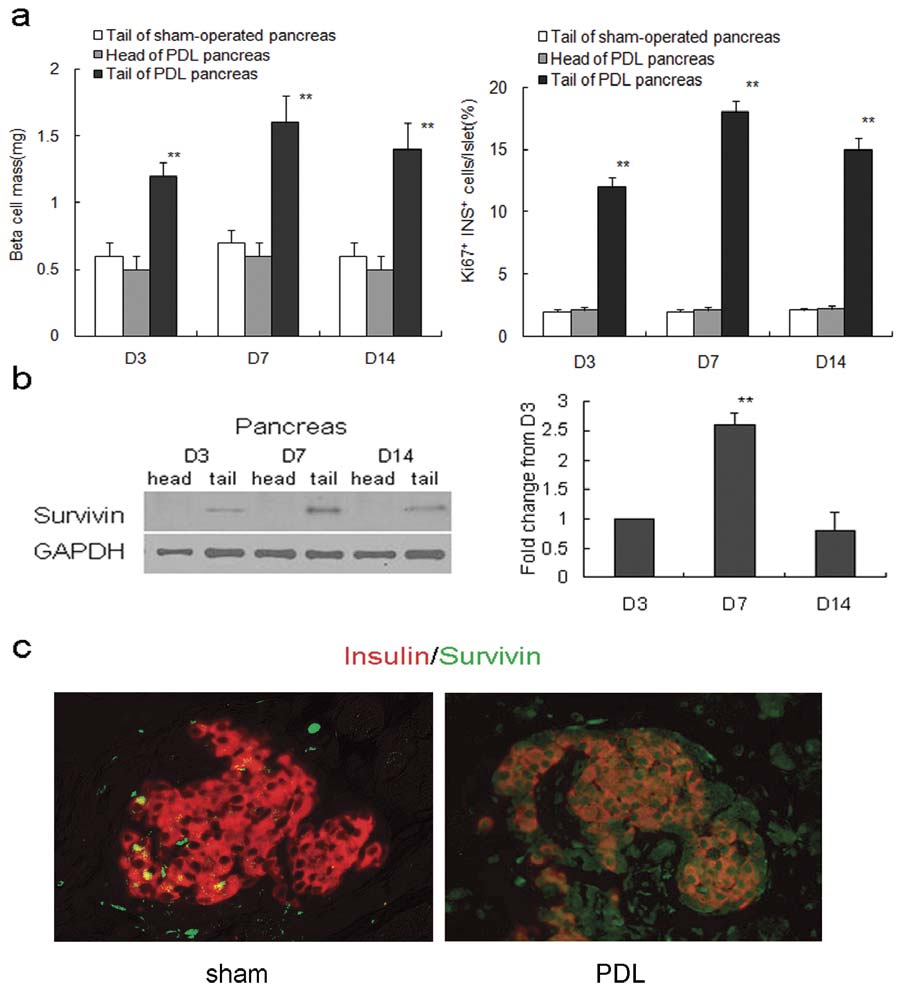
PDL activates survivin expression and promotes beta-cell mass expansion. (a) PDL markedly increased beta-cell mass (left panel) and beta-cell proliferation (right panel) in the tail part of pancreas (black bars) at day 3, 7 and 14 as compared to the unligated head of the same pancreas (gray bars) and the tail of a sham-operated pancreas (white bars) (n = 5 at each time point). **P<0.01. (b) Western blots (left panel) and quantification (right panel) show that survivin expression is detected in the ligated tail of 8-week-old mouse pancreas from PDL day3 to 14 (n = 5 at each time point). Compared to D3, **p<0.01. (c) Immunofluorescent staining shows that survivin is not expressed in the whole pancreas in the tail of day 7 sham-operated pancreas, but reexpressed throughout the pancreatic epithelium including islet beta cells in the tail of pancreas at PDL day 7 (original magnification, ×200). The data represent as means ± SE.

In C57BL/6 mice, the duct leading to the pancreatic tail was closed while the organ’s head located adjacent to the stomach and duodenum remained unaffected. Within two weeks, the beta-cell mass in the ligated part of pancreas increased more than 2-fold following surgery, and beta-cell proliferation increased 9-fold determined by ki67 staining (Fig. 1a). No difference in beta-cell mass and proliferation was found in the non-ligated portion (head) of the pancreas (Fig. 1a).

Previous reports showed survivin to be present in mouse pancreatic islets transiently during embryonic development and neonatal period, and become undetectable by 2-month of age [20], [21]. We found that survivin was induced in the islets of the ligated tail of pancreas within 2 weeks after PDL,reaching its peak at PDL day 7 (Fig. 1b). Immunofluorescent staining showed that survivin was not detectable in the whole pancreas in the tail of day 7 sham-operated pancreas, but regained its expression throughout the pancreatic epithelium including islet beta cells in the tail of pancreas7 days after PDL (Fig. 1c).

### Generation of Beta-cell-specific Survivin-deficient Mice

**Figure 2:**
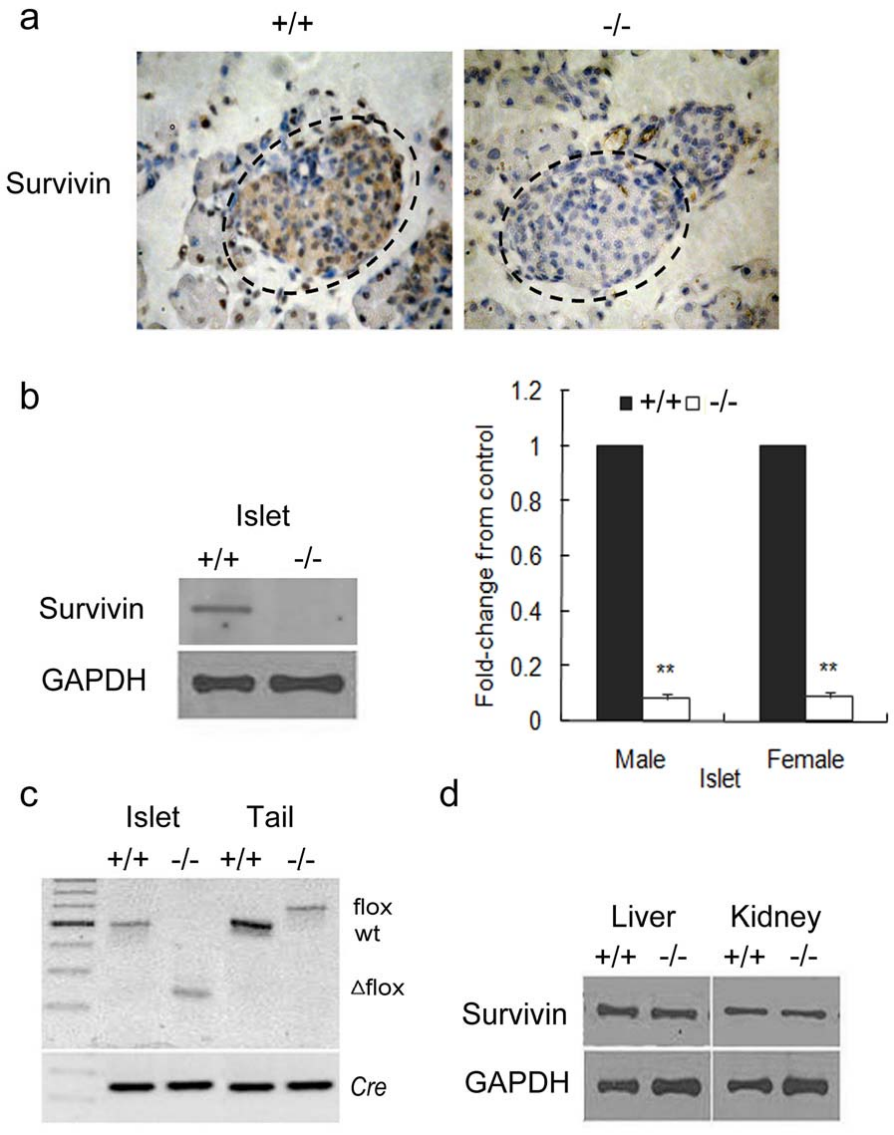
Beta cell-specific deletion of survivin. (a) Immunohistochemistry showing survivin deletion in pancreatic beta cells at P1 from RIPcre^+^survivin^fl/fl^ mice (original magnification, ×400). (b) Western blots (left panel) and quantification (right panel) showing decreased expression of survivin in isolated islets from RIPcre^+^survivin^fl/fl^ mice in both sexes (n = 5 per genotype). Mutant mice, white bars; controls, black bars. (c) PCR analysis of Cre-mediated recombination of the survivin locus (Δflox) in islet and mouse tail DNA. (d) Expression of survivin in the liver and kidney is unchanged. +/+, RIPcre^+^survivin^+/+^ mice; −/−, RIPcre^+^survivin^fl/fl^ mice; wt, wildtype. The data represent as means ± SE. **, p<0.01vs control.

To investigate the essential role of survivin induction in the expansion of pancreatic beta-cell mass, we generated a rat insulin promoter (RIP)-driven survivin knockout mouse by Cre-loxP recombination system. The resultant RIPcre^+^survivin^fl/fl^ mice had deletion of survivin specifically in pancreatic beta cells and efficient deletion in the islets in both sexes (Fig. 2a–d). PCR analyses confirmed the presence of the deleted allele specifically in islets of RIPcre^+^survivin^fl/fl^mice (Fig. 2c). The expression of survivin in other tissues, including the liver and kidney, was unaffected (Fig. 2d).

**Figure 3:**
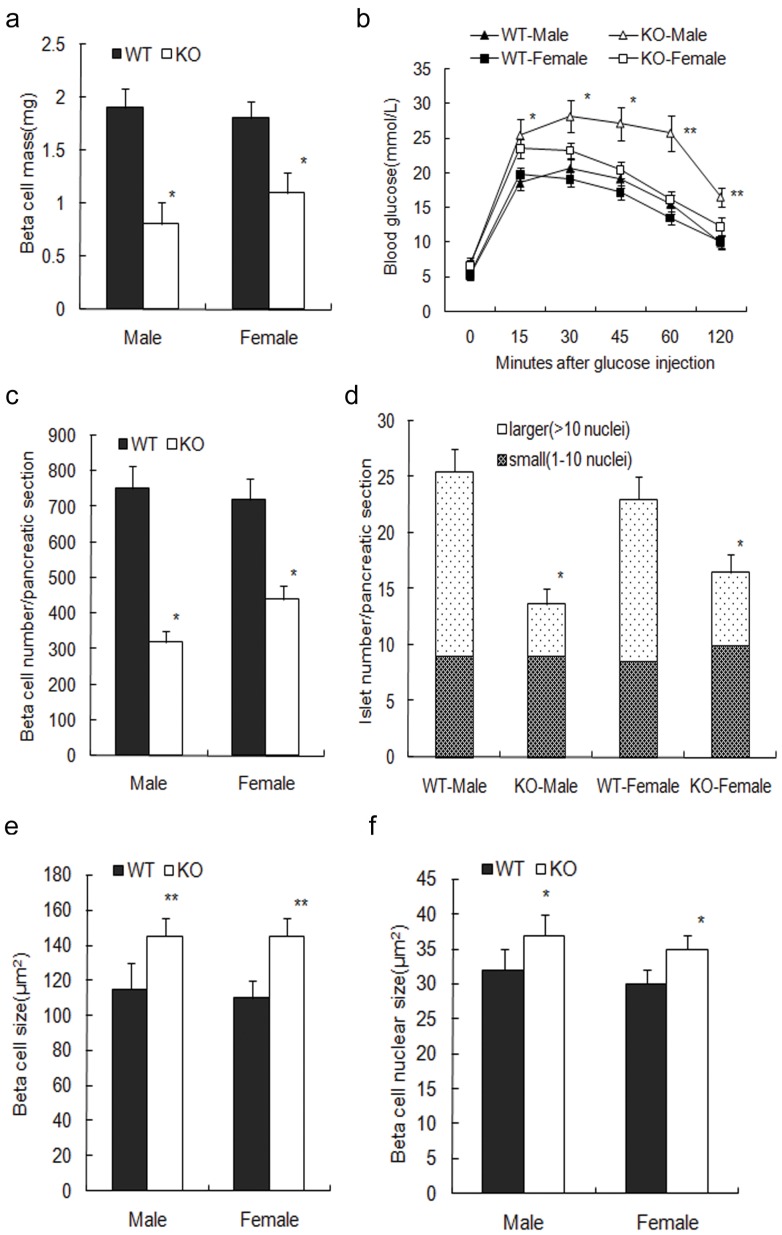
Survivin deletion in beta cells leads to decreased beta-cell mass. (a) Male and female mutant mice exhibit decreased beta-cell mass at 2-month of age as compared to controls (n = 5 per genotype). (b) IPGTTs show that the male mutant mice (△) display higher glucose excursions compared with male controls (▴) whereas the female mutant mice (□) show similar responses as female controls (▪) (n = 8 per genotype). (c) Declined beta-cell number is detected in the male and female mutant mice (n = 5 per genotype). (d) Total islet number is decreased in the mutant mice compared with that in controls in both sexes, mainly due to the decreased definitive islets, with small islets remained similar (n = 5 per genotype). (e) Increased beta-cell size is observed in the mutant mice in both sexes (n = 5 per genotype). (f) Mutant mice exhibit increased beta-cell nucleus size in both sexes (n = 5 per genotype). WT, wildtype (black bars); KO, knockout (white bars). The data represent as means ± SE. *, p<0.05;**, p<0.01vs control.

RIPcre^+^survivin^fl/fl^ mice were born at expected Mendelian frequencies and survived past weaning. Because of the lack of survivin expression during embryonic development and neonatal period, the mutant mice exhibited a decreased beta-cell mass at 2-month of age as compared to controls. The male mutant mice had only about 42% beta-cell mass of that in controls and displayed glucose intolerance during IPGTTs (Fig. 3a,b). However, the female mutant mice still remained glucose tolerant despite an approximate 39% decrease in beta-cell mass (Fig. 3a,b). In both male and female mutant mice, the beta cell size and nucleus size were increased, likely as a compensatory measure (P<0.05, Fig. 3c,e,f).The total islet number was markedly decreased, mainly due to the decrease in the number of larger islets, while small islet number remained similar (P<0.05, Fig. 3d).

### Absence of Survivin in Beta Cells Leads to Glucose Intolerance after PDL

**Figure 4:**
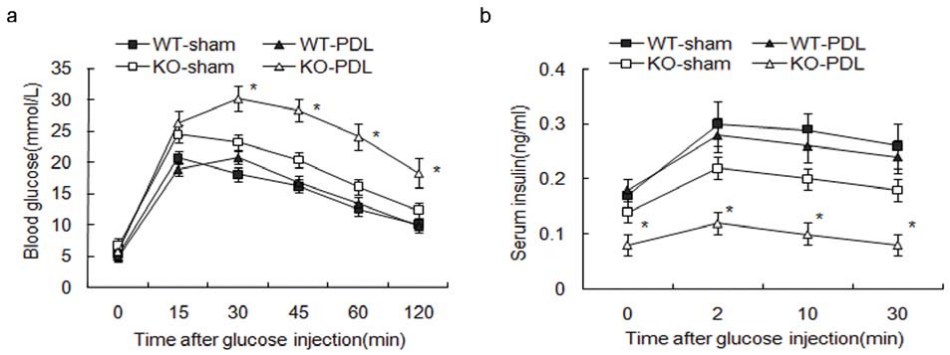
Absence of survivin in beta cells leads to glucose intolerance after PDL. (a) At PDL day 7, female RIPCre^+^Survivin^fl/fl^mice exhibit markedly higher glucose excursions during an IPGTT compared with other groups (n = 10 per genotype). (b) *In vivo* GSIS after i.p. glucose injection is markedly compromised in the mutant mice compared with that in other groups (n = 10 per genotype). ▪, RIPCre^+^Survivin^+/+^ sham day 7; □, RIPCre^+^Survivin^fl/fl^ sham day 7; ▴, RIPCre^+^Survivin^+/+^ PDL day 7; △, RIPCre^+^Survivin^fl/fl^ PDL day 7. The data represent as means ± SE. *, p<0.05 vs sham operation.

To determine whether survivin is necessary for beta-cell expansion following PDL, we subjected female RIPcre^+^survivin^fl/fl^ and control littermates to PDL or sham operation at 2-month of age. IPGTTs were performed at day 7 after PDL or sham operation. Female mice were chosen to eliminate any confounding effects of pre-existing glucose intolerance on beta-cell expansion. The mutant mice exhibited markedly higher glucose excursion compared to controls (Fig. 4a). To further understand this process, we assessed for beta-cell function by GSIS. Insulin secretion in response to an i.p. glucose challenge was markedly compromised in the mutant mice (Fig. 4b), suggesting that glucose intolerance is due to the inadequate supply of insulin from insufficient beta-cell mass.

### Expansion of Beta-cell Mass is Impaired in RIPCre^+^Survivin^fl/fl^ Mice after PDL

**Figure 5:**
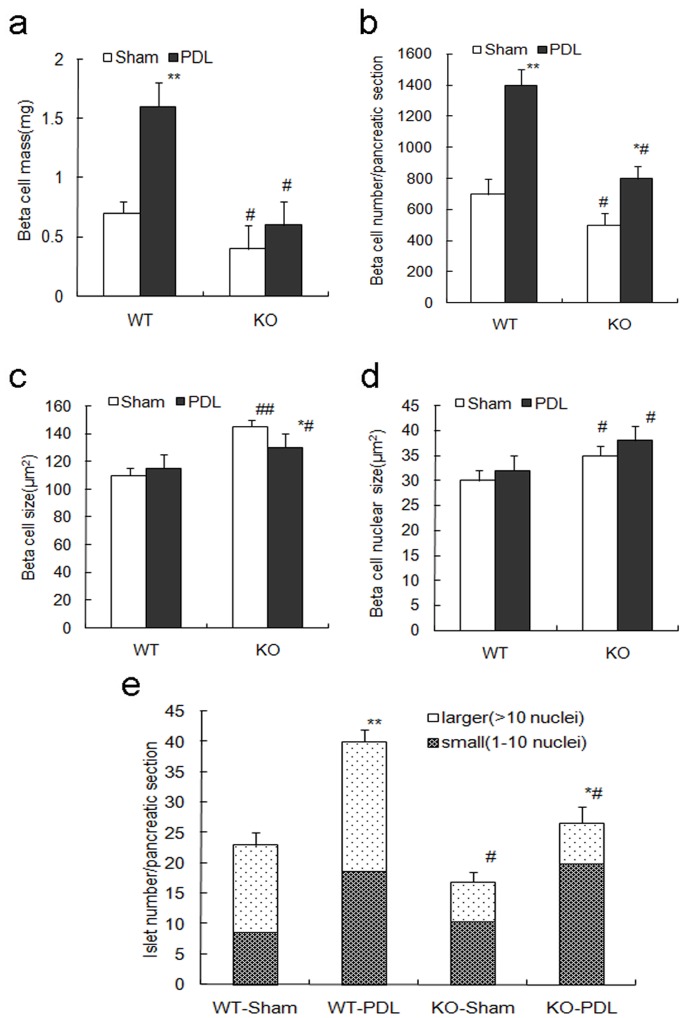
Expansion of beta-cell mass is impaired in RIPCre^+^Survivin^fl/fl^ mice after PDL. (a) At PDL day 7, beta-cell mass expansion in the tail of pancreas in RIPcre^+^survivin^fl/fl^ mice is markedly impaired compared to controls (n = 5 per genotype). (b) The increment of beta-cell number in the pancreas tail at PDL day 7 is markedly attenuated in the mutant mice compared with that in controls (n = 5 per genotype). (c) Beta-cell size is decreased in the pancreas tail of the mutant mice at PDL day 7 compared with that in sham-operated pancreas tail, but is still larger than that in PDL-treated controls (n = 5 per genotype). (d) Mutant mice exhibit increased beta-cell nucleus size in the pancreas tail both in PDL and sham-operated groups (n = 5 per genotype). (e) The increment of total islet number in pancreas tail at PDL day 7 is attenuated in the mutant mice compared to controls, mainly due to the lack of increase in the number of larger islets (n = 5 per genotype). WT, wildtype; KO, knockout. PDL, black bars; sham operation, white bars. The data represent as means ± SE. *p<0.05, **p<0.01 vs sham operation; ^#^P<0.05, ^##^p<0.01 vs control.

To further explore the effects of survivin on beta-cell mass expansion, we examined pancreatic islet morphology after PDL. In wild-type control mice, compared to the sham operation group, beta-cell mass significantly increased by 2.3-fold in the ligated tail of pancreas at day 7 post PDL (P<0.05, Fig. 5a). The individual beta cell size and nucleus size remained similar while a 2-fold increase in beta cell number was observed following the surgery (P<0.01, Fig. 5b-d). Both the small islet and larger islet number was markedly increased within the ligated pancreas tail (P<0.05, Fig. 5e). Small islets likely represented newly formed beta cells from neogenesis whereas increment in larger islets was likely from the replication of preexisting beta cells. So both beta cell neogenesis and self-duplication of existing beta cells might contribute to the expansion of beta-cell mass after PDL.

However, beta-cell mass expansion in the RIPCre^+^Survivin^fl/ fl^mice at day 7 post PDL was significantly impaired compared with that of control wild-type mice (P<0.05, Fig. 5a). Similarly, the increase in beta-cell number was markedly attenuated in the mutant mice (P<0.05, Fig. 5b). The average beta cell size was significantly reduced in the ligated pancreas tail of the mutant mice after PDL compared with sham group, likely due to beta cell division (P<0.05, Fig. 5c). The beta cell nucleus size remained increased in the pancreas tail of the mutant mice compared with that in controls after PDL (P<0.05, Fig. 5d). Although the increment of total islet number was significantly attenuated in the mutant mice, it was attributed mainly by the lack of increase in the number of larger islets while the increment of smaller islets were similar to that of wild-type mice (P<0.05, Fig. 5e), suggesting that survivin deletion impairs islet growth after PDL, rather than new islet formation.

**Figure 6:**
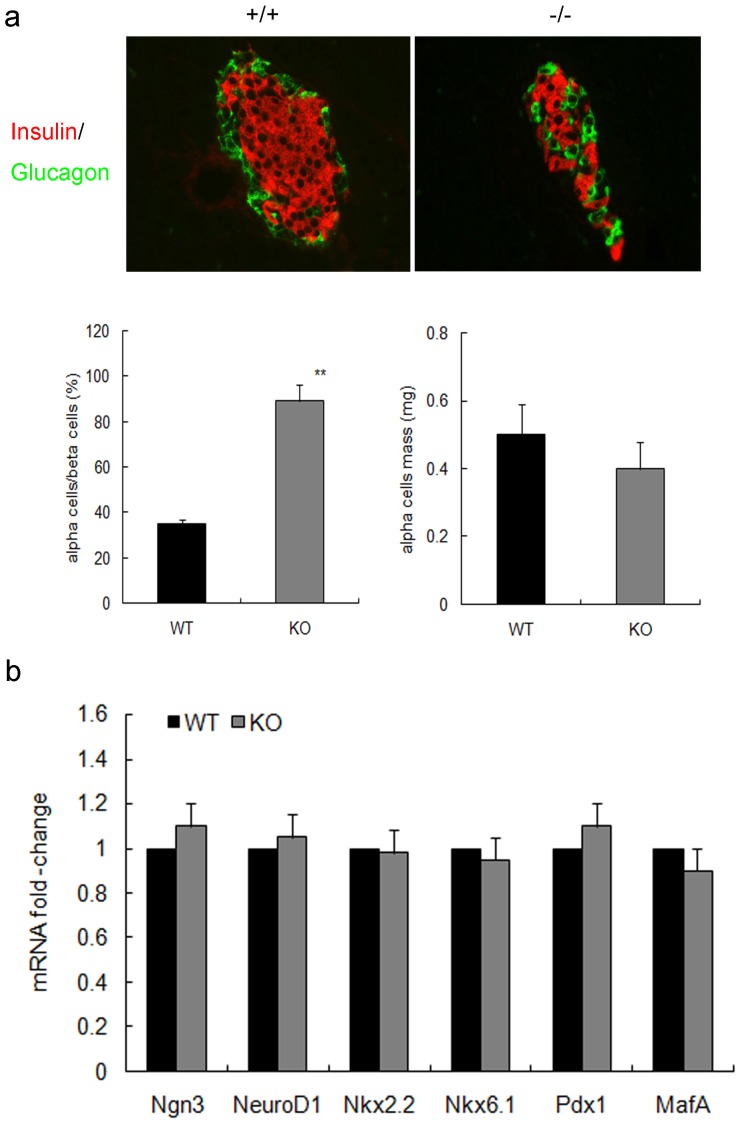
Islet development in RIPcre^+^survivin^fl/fl^ mice and controls after PDL. (a) In day 7 PDL pancreas, insulin and glucagon positive cells show abnormal distribution in the mutant mice (upper panel, original magnification, ×400). Quantification reveals that the knockout islet has a markedly higher proportion of alpha cells to beta cells as compared with controls (lowerleft panel). But alpha cell mass is similar in the two groups (lower right panel) (n = 5 per genotype). (b) Fold change in the expression of the indicated genes related to islet neogenesis as determined by quatitative PCR are similar in day 7 PDL pancreas in the mutant mice (gray bars) compared with controls (black bars) (n = 5 per genotype). WT, wildtype; KO, knockout. The data represent as means ± SE. **p<0.01 vs control.

Our previous data have shown that the decreased beta-cell mass in the mutant mice was not accompanied by abnormal islet development [20]. However, at day 7 post PDL, survivin-deficient islets had an abnormal distribution of alpha and beta cells likely due to impaired beta-cell expansion. The proportion of alpha cells to beta cells was markedly increased in the mutant mice (P<0.01, Fig. 6a). However, alpha cell mass was similar in the two groups. Furthermore, there were no differences in genes expression related to islet neogenesis such as Ngn3, NeuroD1, Nkx2.2, Nkx6.1, Pdx1 and MafA between the two genotypes (Fig. 6b), suggesting that survivin deletion in beta cells did not affect islet neogenesis from endocrine progenitor cells after PDL.

### Cell Proliferation is Defective in Survivin-deficient Betacells after PDL

**Figure 7:**
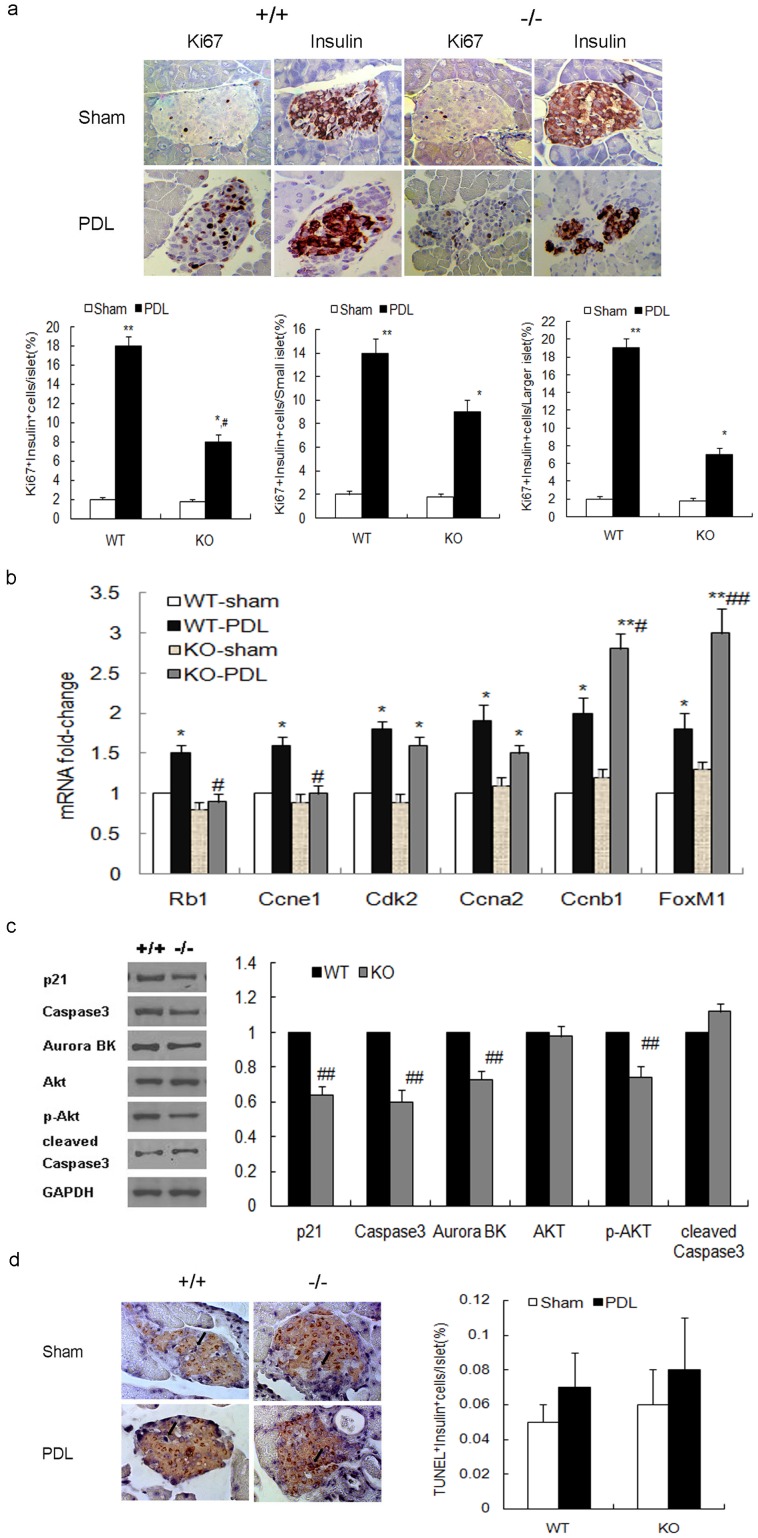
Beta-cell proliferation is partially inhibited in RIPCre^+^Survivin^fl/fl^ mice after PDL. (a) Representative Ki67 and insulin immunostaining on consecutive pancreatic sections (upper panel) and quantification (lower panel) reveal that PDL markedly stimulates beta-cell proliferation in the ligated tail of pancreas both in the mutant mice and controls at day 7. However, the increment is significantly attenuated in the mutant mice as compared to that in controls (left lower panel), especially within larger islets (right lower panel), while the increment is relatively more preserved in small islets (middle lower panel) (original magnification, ×400; n = 5 per genotype). (b) Fold change in the expression of the indicated genes related to cell cycle as determined by quatitative PCR in day 7 PDL or sham-operated pancreas in the mutant mice compared with controls (n = 5 per genotype). (c)Western blots and quantification show a decrease in p21, Caspase-3, Aurora B kinase and phospho-AKT expression in islets of day 7 PDL pancreas in mutant mice (gray bars) compared to controls (black bars) (n = 5 per genotype). (d) Representative insulin and TUNEL co-staining in the ligated tail of pancreas (left panel; original magnification, ×400; apoptotic beta cells are indicated by arrows) and quantification (right panel) show low rate of beta cell apoptosis at day 7 following ligation or a sham operation with no significant difference between the mutant mice and controls (n = 5 per genotype). +/+, RIPcre^+^survivin^+/+^ mice; −/−, RIPcre^+^survivin^fl/fl^ mice. WT, wildtype; KO, knockout. PDL, black bars; sham operation, white bars. The data represent as means ± SE. *p<0.05, **p<0.01 vs sham operation; ^#^P<0.05, ^##^p<0.01 vs control.

To elucidate which specific cellular processes are affected in beta-cell mass expansion in survivin mutant mice after PDL, we first assessed beta-cell proliferation at PDL day 7. In control wildtype mice, PDL dramatically increased beta-cell proliferation by 9-fold in the ligated tail of pancreas compared with the sham-operated group. However, the induction in beta-cell proliferation in RIPcre^+^survivin^fl/fl^ mice was significantly blunted, by 4.4-fold (P<0.05,Fig. 7a). This blunting in the increase of beta cell proliferation in response to PDL appears to be mainly accounted by a defect in proliferation of larger islets. In contrast, proliferation of small islet beta cells was better preserved following PDL compared to sham operation. These data further suggest that replication of preexisting beta cells is largely survivin-dependent.

To attempt to gain insight into potential cell cycle proteins abnormalities, we first performed quantitative PCR on RNA from day 7 PDL or sham-operated pancreas for genes involved in cell cycle progression. Compared with sham-operated groups, there was a marked increase in the expression of Rb1, Ccne1, Cdk2, Ccna2, Ccnb1 and forkhead box M1 (FoxM1) in wildtype mice after PDL. However, compared with wildtype controls, there was an attenuation in the rise of Rb1, Ccne1, Cdk2, and Ccna2 in response to PDL in the survivin mutant mice. On the other hand, there was a significant further increase in expression of Ccnb1 and FoxM1 in survivin mutant mice (Fig. 7b). The attenuation in the rise of Rb1 gene expression could reflect a compensatory downregulation in an attempt to enhance G1/S transition in cell cycle [25]. The upregulation of FoxM1 and Ccnb1 gene expression could also be adaptive changes in an attempt to promote cell cycle progression [26]. We further explored the expression of mitosis-related proteins in the islets of ligated pancreas tail. While p21 is mostly known as a cell cycle inhibitor, it can also promote mitosis by forming a complex with Caspase-3 and survivin [27]. Aurora B kinase binds to survivin to form the chromosomal passenger complex. In the absence of survivin, a decrease in p21, Caspase-3 and Aurora B kinase expression islets were observed after PDL, which may lead to defective mitosis (Fig. 7c). Our findings support the hypothesis that survivin is required for beta-cell proliferation in adults after PDL through cell cycle regulation. Moreover, p-Akt was significantly decreased in islets of mutant mice (Fig. 7c), indicating that survivin may also regulate the phosphatidylinositol 3-kinase-Akt signaling pathway which plays an important role in beta-cell proliferation.

We also examined beta-cell apoptosis in the ligated tail of pancreas at PDL day 7. The rate of beta-cell apoptosis was low and not significantly different between the mutant mice and control wild-type mice after PDL or sham operation (Fig.7d). Furthermore, we did not detect any significant changes in cleaved Caspase-3 in the islets of mutant mice compared with controls (Fig. 7d), indicating that apoptosis did not significantly contribute to the defect in islet expansion in survivin-deficient beta cells after PDL.

## Discussion

Survivin is highly expressed within pancreatic islets during embryogenesis and neonatal period, however eventually becomes undetectable by 2-month of age [20], [21]. We previously showed that mice with survivin deleted throughout the pancreatic epithelium exhibit impaired beta-cell mass establishment and postnatal remodeling secondary to reduced beta-cell proliferation with perturbations in cell cycle proteins [20]. Here, we show that survivin expression increases in the islets of the ligated tail of adult mouse pancreas as early as three days post PDL, which was correlated with increased beta-cell proliferation and mass expansion. In contrast, RIPcre^+^survivin^fl/fl^ mice with specific deletion of survivin in beta cells showed impaired beta-cell mass expansion following PDL due to defective beta-cell proliferation.

PDL is a normoglycemic pancreatic injury model that stimulates beta-cell mass expansion by both beta-cell neogenesis from endogenous progenitors and self-duplication of preexisting beta cells in adult pancreas [5], [22]. We found that PDL led to resurgence of survivin expression in the islets of the ligated tail of adult mouse pancreas within 2 weeks during a period of enhanced beta-cell proliferation. A recent study in mice has shown that FoxM1 is partially required for proliferation of preexisting beta cells stimulated by 60% partial pancreatectomy (PPx) [28]. Survivin, as the known FoxM1 target, was the most significantly upregulated gene during the peak of beta-cell proliferation following PPx. Given the multi-regulatory role of survivin in the various cellular processes, our result demonstrated that survivin was required for injury-stimulated beta-cell proliferation in adult mice.

To dissect the physiological role of survivin in beta-cell mass expansion, we generated RIP-driven survivin knockout mice. Because of the lack of survivin expression during embryonic development and neonatal period, the mutant mice exhibited a decreased beta-cell mass at 2-month of age compared to wildtype littermate controls. The male mutant mice displayed glucose intolerance whereas the female mutant mice remained glucose tolerant despite the reduced beta-cell mass. This phenomenon has been observed in other rodent models of diabetes [29]–[31] and has been linked to a protective effect of estrogen [32], [33]. We took advantage of this finding and focused on female mice, as this would avoid the confounding effects of preexisting defects in glucose homeostasis. The female mutant mice indeed exhibited glucose intolerance, compromised beta-cell mass expansion, blunted increase in islet and beta-cell number, and impaired beta-cell proliferation following PDL compared to control wildtype littermates after the same procedure.

Importantly, PDL-stimulated beta-cell proliferation was blunted, but not completely abolished within the ligated tail of the pancreas in the mutant mice. This may be due to beta-cell neogenesis. PDL has been shown to activate the progenitors in the ductal lining to differentiate in Ngn3 dependent way and give rise to glucose responsive beta cells that subsequently proliferate to increase the functional beta-cell mass [5]. Gene expressions related to islet neogenesis such as Ngn3, NeuroD1, Nkx2.2, Nkx6.1, Pdx1 and MafA are similar between the mutant mice and controls, suggesting that survivin deletion in beta cells does not impair islet neogenesis after PDL. Although the increment of total islet number was markedly attenuated in RIPcre^+^survivin^fl/fl^ mice compared to wildtype littermates 7 days after PDL, this was largely due to impairment in the rise in the number of larger islets. In contrast, rise in the number of smaller islets were relatively preserved, thereby not significantly contributing to the defect of islet mass expansion observed in mutant mice. These data support our observation that small islets represent newly formed beta cells that likely do not require survivin for its formation or proliferation, whereas expansion of already-formed islets that arise from replication of preexisting beta cells appears to require survivin. The similar phenomenon was found in the FoxM1 knockout mice after 60% PPx [28]. FoxM1 was not required for proliferation of beta cells within small endocrine cell clusters located in the regenerating portion of the pancreas but was specifically required for proliferation of preexisting beta-cells within largerislets. FoxM1 transactivates genes that coordinate the G1/S and G2/M transitions, karyokinesis and cytokinesis [26]. As the known FoxM1 target, survivin fits well within the molecular pathway downstream of Foxm1 and likely functions to assist them in the modulating of beta-cell replication. In our study, FoxM1 gene expression was upregulated by 1.7 fold in survivin mutant mice after PDL compared with wild-type controls. However, defect in beta-cell proliferation indicated that the cell cycle promotion effect of FoxM1 might be mediated through survivin-dependent pathway.

Survivin plays an important role in cell cycle regulation. During G1, survivin transcription increases and reaches a peak in G2/M [34], [35]. During mitosis, survivin has been shown to co-localize with caspase-3 and p21 at the centrosome [36]. As a chromosomal passenger protein, survivin is involved in chromosome segregation, central spindle formation, cytokinesis and spindle checkpoint maintenancethrough its interactions with other passenger proteins, inner centromere protein and AuroraB kinase [37]. Thus, disruption of survivin may lead to abnormal centrosome duplication and formation of multipolar spindles, multinucleation and polyploidy. In keeping with these observations, our *in vivo* data reveal that survivin-deficient beta cells have many enlarged nuclei, together with decreased expression of p21, Caspase-3 and AuroraB kinase. The decreased gene expression of Rb1 and Ccne1 could cause the delay on G1/S transition during cell cycle. In addition, levels of phosphorylated Akt were significantly decreased in the mutant islets, indicating that the phosphatidylinositol 3-kinase–AKT pathway may be involved in survivin mediated beta-cell proliferation. Therefore, resurgence of survivin in response to PDL may be critical in recruiting more beta cells to enter cell cycle that is necessary for islet expansion.

Taken together, our data show that resurgence of survivin expression in the pancreatic beta cells after PDL is essential for beta-cell mass expansion largely through beta-cell proliferation. The preexisting beta cells seemingly exhibit a stronger requirement for survivin than new beta cells formed by neogenesis. This study highlights the importance of survivin-dependent replication of preexisting beta cells as a mechanism of beta-cell mass expansion. Given that beta cell expansion appears to occur through proliferation of existing beta cells, survivin may indeed be a viable therapeutic target for beta cell expansion in diabetes.

## Supporting Information

Table S1 **Primers for qRT-PCR.**
(DOC)
