# Supplementary material for: Survivin Is Required for Beta-Cell Mass Expansion in the Pancreatic Duct-Ligated Mouse Model
Source: PLoS One. 2012 Aug 1;7(8):e41976. doi: 10.1371/journal.pone.0041976 (PMC3411579; doi:10.1371/journal.pone.0041976)
Supplement: Table S1 — Primers for qRT-PCR. (DOC) [file pone.0041976.s001.doc]

**Supplemental Table S1.**

**Supplemental Table S1**

**Primers for qRT-PCR.**

| Transcript | Forward Primer | Reverse Primer | GeneBank accession no. | Size of PCR product (bp) |
| --- | --- | --- | --- | --- |
| Ngn3 | 5’- TGACCCTATCCACTGCTGCTT -3’ | 5’- CCTCATCCACCCTTTGGAGTT -3’ | NM_009719 | 104 |
| Neurod1 | 5’- GCCCAGCTTAATGCCATCTTT -3’ | 5’- CAAAAGGGCTGCCTTCTGTAA -3’ | NM_010894 | 113 |
| Nkx2-2 | 5’- TCGCTCTCCCCTTTGAACTTT -3’ | 5’- GTTAACGTTGGGATGGTTTGG -3’ | NM_010919 | 120 |
| Nkx6-1 | 5’- AACACACCAGACCCACGTTCT -3’ | 5’- ATCCCCAGAGAATAGGCCAAG -3’ | NM_144955 | 115 |
| Pdx1 | 5’- CTTAACCTAGGCGTCGCACAA -3’ | 5’- GAAGCTCAGGGCTGTTTTTCC -3’ | NM_008814 | 103 |
| MafA | 5’- GAGGAGGTCATCCGACTGAAA -3’ | 5’- GCACTTCTCGCTCTCCAGAAT -3’ | AB086961 | 114 |
| Rb1 | 5’-TGCATCTTTATCGCAGCAGTT-3’ | 5’-GTTCACACGTCCGTTCTAATTTG-3’ | [NM_009029](http://www.ncbi.nlm.nih.gov/nuccore/NM_009029.2) | 208 |
| Ccne1 | 5’-GTGGCTCCGACCTTTCAGTC-3’ | 5’-CACAGTCTTGTCAATCTTGGCA-3’ | [NM_007633](http://www.ncbi.nlm.nih.gov/entrez/query.fcgi?cmd=Search&db=Nucleotide&term=NM_007633) | 101 |
| CDK2 | 5’-CCTGCTTATCAATGCAGAGGG-3’ | 5’-TGCGGGTCACCATTTCAGC-3’ | [NM_016756](http://www.ncbi.nlm.nih.gov/entrez/query.fcgi?cmd=Search&db=Nucleotide&term=NM_016756) | 203 |
| Ccna2 | 5’-GCCTTCACCATTCATGTGGAT-3’ | 5’-TTGCTGCGGGTAAAGAGACAG-3’ | [NM_009828](http://www.ncbi.nlm.nih.gov/entrez/query.fcgi?cmd=Search&db=Nucleotide&term=NM_009828) | 118 |
| Ccnb1 | 5’-AAGGTGCCTGTGTGTGAACC-3’ | 5’-GTCAGCCCCATCATCTGCG-3’ | [NM_172301](http://www.ncbi.nlm.nih.gov/entrez/query.fcgi?cmd=Search&db=Nucleotide&term=NM_172301) | 228 |
| Foxm1 | 5’-CACTTGGATTGAGGACCACTT-3’ | 5’-GTCGTTTCTGCTGTGATTCC-3’ | NM_008021.4 | 226 |
| GAPDH | 5’- CCTGGAGAAACCTGCCAAGTA -3’ | 5’- TGGAAGAGTGGGAGTTGCTGT -3’ | NM_008084 | 137 |
